# Stiffness of sphere–plate contacts at MHz frequencies: dependence on normal load, oscillation amplitude, and ambient medium

**DOI:** 10.3762/bjnano.6.87

**Published:** 2015-03-30

**Authors:** Jana Vlachová, Rebekka König, Diethelm Johannsmann

**Affiliations:** 1 Clausthal University of Technology, Institute of Physical Chemistry, Arnold-Sommerfeld-Straße 4, 38678 Clausthal-Zellerfeld, Germany

**Keywords:** contact mechanics, contact splitting, contact stiffness, partial slip, quartz crystal microbalance

## Abstract

The stiffness of micron-sized sphere–plate contacts was studied by employing high frequency, tangential excitation of variable amplitude (0–20 nm). The contacts were established between glass spheres and the surface of a quartz crystal microbalance (QCM), where the resonator surface had been coated with either sputtered SiO_2_ or a spin-cast layer of poly(methyl methacrylate) (PMMA). The results from experiments undertaken in the dry state and in water are compared. Building on the shifts in the resonance frequency and resonance bandwidth, the instrument determines the real and the imaginary part of the contact stiffness, where the imaginary part quantifies dissipative processes. The method is closely analogous to related procedures in AFM-based metrology. The real part of the contact stiffness as a function of normal load can be fitted with the Johnson–Kendall–Roberts (JKR) model. The contact stiffness was found to increase in the presence of liquid water. This finding is tentatively explained by the rocking motion of the spheres, which couples to a squeeze flow of the water close to the contact. The loss tangent of the contact stiffness is on the order of 0.1, where the energy losses are associated with interfacial processes. At high amplitudes partial slip was found to occur. The apparent contact stiffness at large amplitude depends linearly on the amplitude, as predicted by the Cattaneo–Mindlin model. This finding is remarkable insofar, as the Cattaneo–Mindlin model assumes Coulomb friction inside the sliding region. Coulomb friction is typically viewed as a macroscopic concept, related to surface roughness. An alternative model (formulated by Savkoor), which assumes a constant frictional stress in the sliding zone independent of the normal pressure, is inconsistent with the experimental data. The apparent friction coefficients slightly increase with normal force, which can be explained by nanoroughness. In other words, contact splitting (i.e., a transport of shear stress across many small contacts, rather than a few large ones) can be exploited to reduce partial slip.

## Introduction

**Figure 1:**
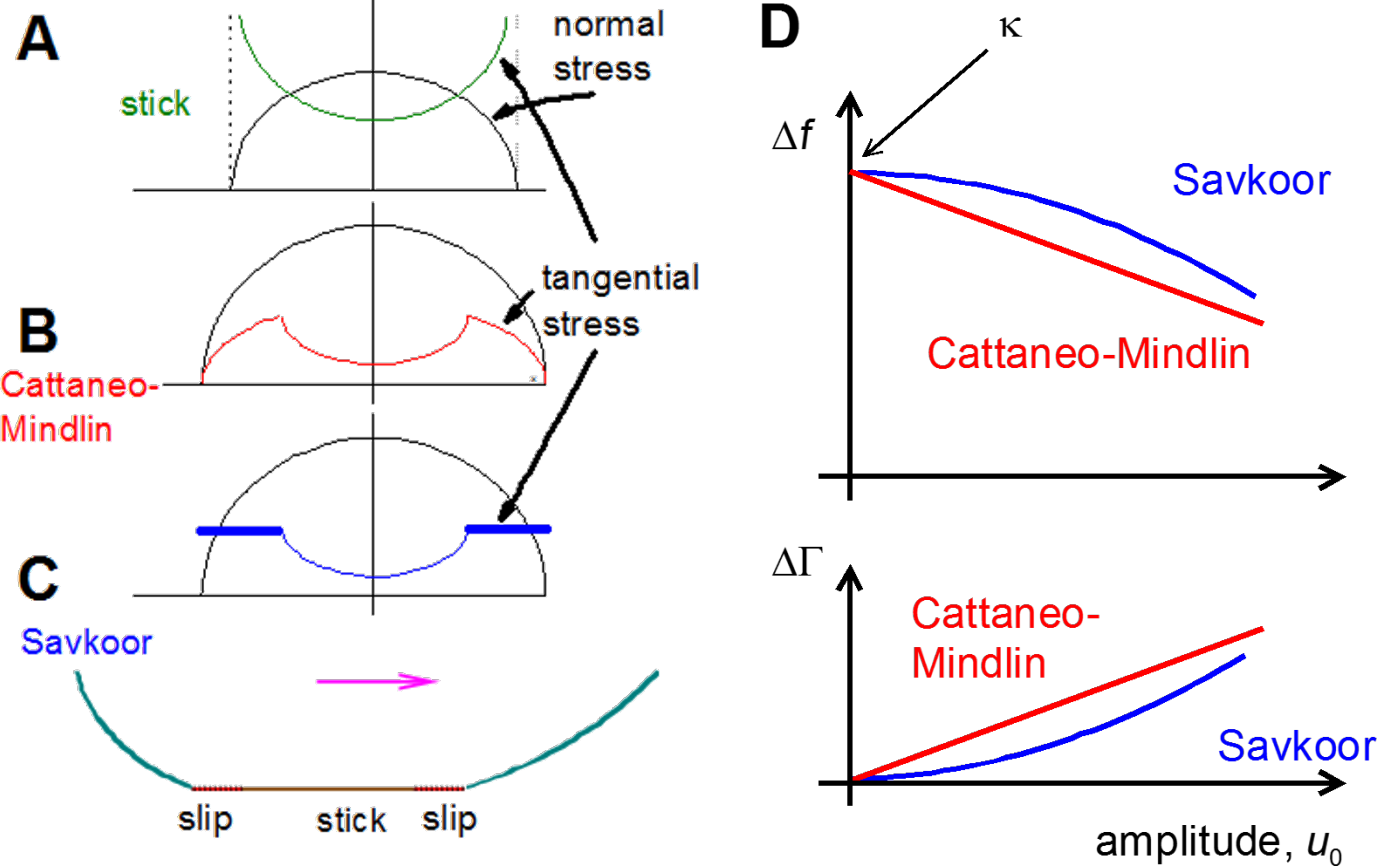
Sketch of the mechanisms underlying partial slip. A Hertzian contact under a tangential load has infinite tangential stress at the edge of the contact (A). This singularity is removed by allowing for slip in a circular region close to the edge. In CM theory, the tangential stress in the sliding region is proportional to the normal stress, where the latter follows the Hertz model (B). In an alternative model formulated by Savkoor, the tangential stress in the sliding region is constant (C). When probing such contacts with the contact resonance method, the two models lead to different dependences of the shifts in frequency, Δ*f*, and shifts in bandwidth, ΔΓ, on the amplitude, *u*_0_. Δ*f* and ΔΓ depend linearly and quadratically on amplitude for the CM model and the Savkoor model, respectively (D).

**Figure 2:**
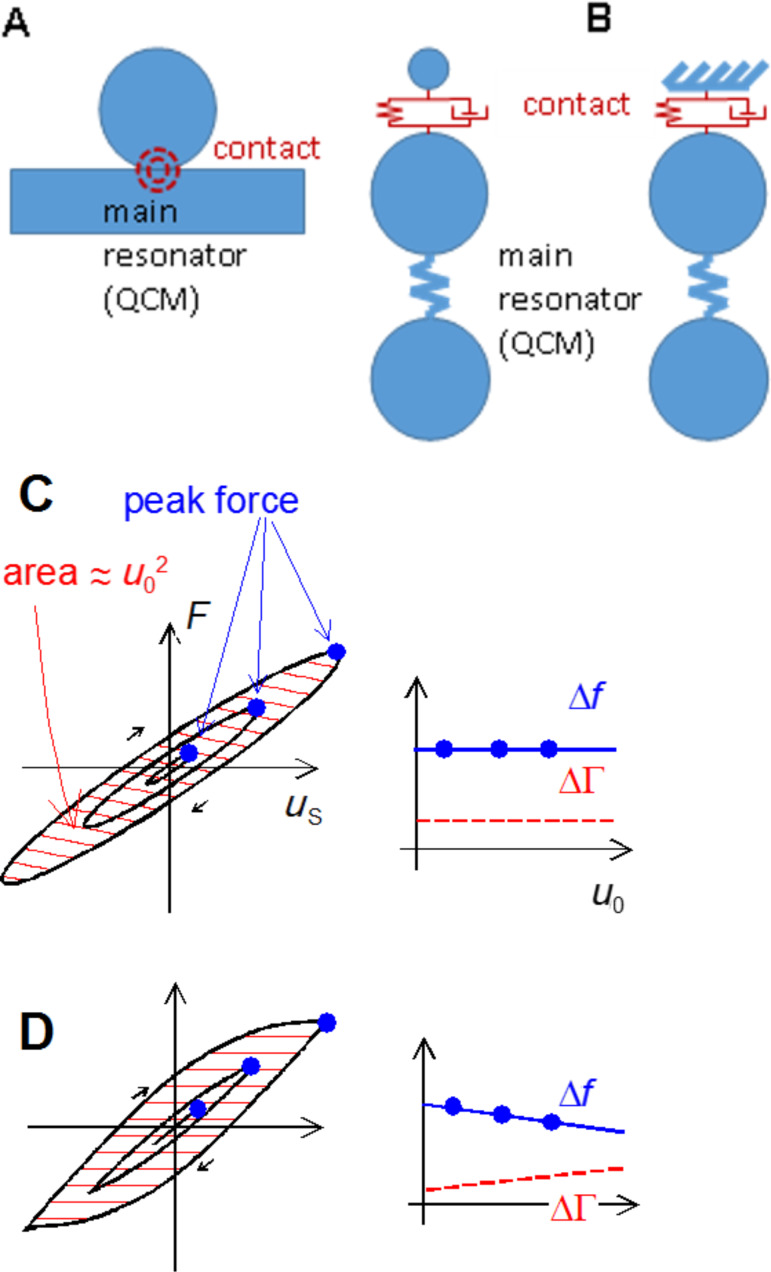
(A) For a narrow contact between a sphere and a plate, deformation occurs close to the contact, only. The contact may be depicted as a spring, or more generally, as a Voigt element, where the latter also accounts for viscous dissipation (B). In the case, where the sphere is heavy enough to be clamped in space by inertia, it can be depicted as a wall (right-hand side in Β). (C,D): Illustration of how Δ*f* and ΔΓ depend on the shape of the force–displacement loop. (C): viscoelastic contact and (D): partial slip according to Cattaneo and Mindlin. The frequency shift is roughly proportional to the ratio of force and displacement at the turning point (full dots). ΔΓ is proportional to the area inside the loop divided by *u*_0_^2^ (hatched). (C) and (D) adapted with permission from [10], copyright 2013 the American Physical Society.

Partial slip is a widespread and multifacetted phenomenon. When a contact experiences partial slip, parts of a contact stick to each other under a tangential stress, while others slide. Partial slip is found in many tribological situations of practical relevance. This includes fretting wear [1–2] granular media [3], earthquakes [4], and the collision between particles [5]. Early models of partial slip were formulated independently by Cattaneo [6] and Mindlin [7], who were concerned with a Hertzian contact. If the entire contact area sticks, a continuum treatment predicts a stress singularity at the rim of the contact (Figure 1A). However, infinite stress is unrealistic, and among the mechanisms removing the singularity is partial slip. Partial slip implies that those areas, where the tangential stress exceeds a certain critical value, slide and thereby lower the local stress. Cattaneo and Mindlin assumed that the frictional stress in the sliding zone, σ, is proportional to the normal pressure, *p*, as in Coulomb friction (Figure 1C). The ratio of σ and *p* is the friction coefficient, µ. From the Cattaneo–Mindlin (CM) model, one can derive predictions for the width of the sliding region (which is of annular shape) and for the force–displacement relation (Figure 2D below) [8–9].

Partial slip as such is an accepted and frequently observed phenomenon. The details of the CM model, however, are being debated for a variety of reasons. Etsion [11] gives a detailed account. The first category of problems originates from the numerous assumptions in the formulation of the model. For example, the normal pressure is assumed to stay constant during tangential loading. A second set of limitations is related to the idealized conditions. The CM model ignores roughness, capillary forces, plastic deformation, and the effects of contamination. In particular, plastic deformation can lead to junction growth, which stiffens the contact rather than weakening it [12–13].

There is a particular shortcoming that is on the one hand widely observed, but also easily fixed on a heuristic level on the other. The CM model ignores viscous dissipation. In consequence, the energy dissipated in reciprocating sliding scales as the cube of the oscillation amplitude in the low-amplitude limit. Following from this scaling law, the damping of a resonator, which experiences particle slip in one way or another, should go to zero at small amplitudes. An explanation of the contact resonance method, which probes these relations, is given below. Deviating from this scaling prediction, the contacts usually do damp a resonance even at the smallest accessible amplitudes. This type of damping must be related to linear viscoelasticity, meaning that the corresponding stresses are proportional to displacement (Figure 2C). While such viscous processes are not contained in the CM model, they can be added into it in an ad hoc manner (see Equation 1).

In the Results and Discussion section, we address a further rather fundamental criticism of the CM model, which starts out from the extent to which a macroscopic view of friction guides its formulation. Macroscopic concepts enter the CM model at two separate instances. Firstly, a sliding stress proportional to the normal pressure is commonly associated with Coulomb friction. In Coulomb friction, the tangential force is related to the actual area of contact, to be distinguished from the nominal area of contact due to surface roughness. These arguments should not apply on the nanoscale. Savkoor has responded to this criticism with a modified model of partial slip, which assumes the tangential stress in the sliding zone to be constant, independent of normal pressure [14–15]. The value of the constant stress, τ_0_, is the free parameter of the model. Savkoor solved the equations of continuum elasticity and derived the force–displacement relations. These relations differ from the CM model in the details, but deciding between the two models based on the shape of the force–displacement loop is somewhat of a challenge. Interestingly, it is rather easy to distinguish between the Savkoor model and the Cattaneo–Mindlin model with the contact resonance method because the Cattaneo–Mindlin model predicts a linear dependence of frequency and bandwidth on amplitude, while the same relations are parabolic if derived from the Savkoor model. This difference is easily observed in experiment [16].

A second element of the CM model of genuinely macroscopic nature is the notion of a stress singularity at the edge of the contact. It is essential that this peak in stress at the edge is indeed strong enough to locally initiate sliding. Gao and Yao have mathematically analyzed a related problem, namely the detachment of a fiber end from a flat surface under tensile load [17]. Such a contact displays a peak in tensile stress at the edge, which governs the pull-off force if the contact diameter is larger than about 100 nm. Pull-off then results in crack propagation. Partial slip in the Cattaneo–Mindlin sense also results in crack propagation, where the modes of crack opening are II and III, as opposed to mode I, which operates during pull-off [18]. Gao and Yao find that the crack propagation mechanism becomes inefficient once the contact diameter falls to below 100 nm. For small contacts, the stress concentration at the edge becomes less and less significant. Translated to the tangential load problem, the analysis by Gao and Yao shows that the transition from stick to slip may occur by crack propagation (that is, by partial slip in the Cattaneo–Mindlin sense), but that small contacts may also start to slide as a whole. Even if partial slip at individual contacts is found, it is expected to be more prominent for larger contacts because the maximum level of stress depends on the ratio of the contact diameter to the radius of the crack tip.

From an engineering perspective, partial slip (also called microslip) has a slightly different meaning. It mostly denotes a small tangential displacement at contacts between rough surfaces. These small displacements per se have little influence on the strength of the contact. They are still of immense practical relevance because they cause fretting wear [19–21], which is a special type of corrosion. Microslip at multicontact interfaces is different from partial slip in the Cattaneo–Mindlin sense because it involves a debonding of the weakly coupled load–bearing asperities and, also, because new contacts can form at large relative displacements [22]. Depending on the distance between the individual load–bearing asperities, these are elastically coupled to each other [23]. If the contacts are tightly coupled, there is crack propagation with a peak in stress at the crack tip. Otherwise, the analysis should be based on an ensemble of contacts with a distribution in contact stiffness and contact stress. Bureau et al. have provided such a model [24], making extensive use of the Greenwood–Williamson formalism [25].

The experiments below rely on the contact resonance method. The contact resonance method is also applied on the macroscopic scale [26] and in AFM-based metrology [27]. In particular, the mathematics is closely related to what was reported in [28] and [29]. Differing from many experiments performed with AFM [30–31], the contacts here have a substructure and it is this substructure, which gives rise to the phenomena under discussion. Also, hysteresis is more important in QCM experiments than in AFM experiments. A contact is established between a resonator (which is a quartz crystal microbalance here and is the cantilever in AFM experiments) and an external object. The geometry is configured such that the contact does not overdamp the resonance, but rather shifts the resonance frequency and the resonance bandwidth by small amounts (termed Δ*f* and ΔΓ below). The contact resonance method is well suited to detect nonlinear force–displacement relations because nonlinear behavior leads to a dependence of Δ*f* and ΔΓ on amplitude, *u*_0_, while such a dependence is absent when the system obeys a linear response. Partial slip results in a nonlinearity and whether or not a contact undergoes partial slip can therefore be inferred from the dependence of Δ*f* and ΔΓ on the amplitude. More quantitatively, the Cattaneo–Mindlin model predicts Δ*f* and ΔΓ to scale linearly with *u*_0_ in the low-amplitude limit and this prediction can be tested easily.

The experiments were undertaken with a quartz crystal microbalance (QCM). The QCM is mostly known as a device for thickness determination, but it can equally well be employed to measure contact stiffness. In this regard, it is helpful to view the QCM as a shear wave reflectometer. The amplitude and the phase of the wave reflected at an interface is related to the stiffness of this interface. Acoustic reflectometry was used to measure contact stiffness as early as 1971 [32]. The work reported below is concerned with discrete contacts (as opposed to a multicontact interface), but the physical picture is closely analogous to what is developed in [32]. The presence of contacts at a resonator surface changes the reflectivity of the resonator surface and thereby changes the resonator’s frequency and its bandwidth [26].

There is a different (but equivalent) way of explaining the measurement principle. The resonator can be represented by a lumped element circuit [33], as shown in Figure 2B. The main resonator is at the bottom. Its resonance frequency is given as 2π(κ_R_/*M*_R_)^1/2^ where κ_R_ is the effective stiffness and *M*_R_ is the effective mass. The sample is the small sphere at the top. Because the contact zone is small (Figure 2A), it can be represented by a spring and a dashpot arranged in parallel (a Voigt element). If the resonator is coated with a rigid thin film (or with nanoparticles rigidly attached to the surface), this load increases the resonator’s effective mass, thereby lowering the resonance frequency. In the lumped element representation, this amounts to the sphere at the top in Figure 2B being small and the spring being stiff. Applied in this mode, the QCM determines the value of the effective mass, hence the name “microbalance”. However, millimeter-sized spheres such as the ones studied here are not samples of this kind. They are so heavy that they do not follow the resonator’s MHz motion, but rather are clamped in space by inertia [34]. In the lumped element representation, they are depicted as a wall, attached to the surface across a spring and a dashpot (a Voigt element). It is essential that the contact diameter is much smaller than both the sphere diameter and the wavelength of sound. The deformation is then localized; the bulk of the sphere remains undeformed. The force follows from integration of the stress distribution over the contact area; the displacement is evaluated in the undeformed regions far outside the contact zone. The ratio of force and displacement is the contact stiffness. As we show in the modeling section, the spring constant and the dashpot’s drag coefficient can be easily determined from the shifts of frequency and bandwidth. The ratio of the two represents the loss tangent.

The representation of the contact as a Voigt element only holds as long as the contact behaves linearly. Partial slip, however, results in a nonlinear behavior. Even in the precense of partial slip, one can use the lumped element representation for the sake of an intuitive understanding. Roughly speaking, the apparent contact stiffness decreases at elevated amplitudes because the sticking portion of the contact decreases. The “apparent contact stiffness” here is the stiffness as derived from the frequency shift (Equation 2 below). This intuitive picture can be backed up with a rigorous mathematical model. We briefly recapitulate the mathematics in the modeling section.

In previous work [10], we have reported details of the experimental setup and elaborated on the mathematical details of what the Cattaneo–Mindlin model and the Savkoor model predict for the functions Δ*f*(*u*_0_) and ΔΓ(*u*_0_). The authors in [10] focused on how the amount of partial slip depends on contact size. For the current work, the sphere size was chosen large enough to always guarantee partial slip. An improved experimental setup allowed for a detailed quantitative analysis in both the linear and the nonlinear regime. All experiments were repeated 9 times, which allows for a robust analysis of statistical errors. Finally, we compare experiments undertaken in air to experiments using the same sample, but immersed in water.

## Experimental

### Modeling

#### A QCM loaded with discreate contacts: linear and nonlinear regime

We first consider the viscoelastic contact. According to the small-load approximation, the complex frequency shift at small amplitude is given as [35–36]

[3]
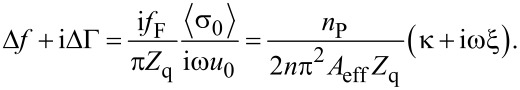


Δ*f* and ΔΓ are the shifts of the frequency and the half-bandwidth at half-height, respectively. The parameter Γ is related to the dissipation factor, *D*, by *D* = Γ/(2*f*). *f*_F_ is the fundamental frequency, which is often 5 MHz. *Z*_q_ = 8.8 × 10^6^ kg∙m^−2^s^−1^ is the shear wave impedance of AT-cut quartz. 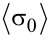 is the area-averaged complex amplitude of the tangential stress at the resonator surface, and *u*_0_ is the amplitude of oscillation. The ratio of stress and velocity (where the latter is equal to iω*u*_0_) is the complex load impedance, *Z*_L_. In the second step in Equation 3, the stress was converted to force by area. The force, in turn, was expressed as tangential stiffness times amplitude (that is, as κ*u*_0_). *n* is the overtone order, *n*_P_ is the number of spheres, and *A*_eff_ is the acoustically effective area (similar to the electrode area, *A*_eff_ can be derived from the experimental data [10]). κ is the tangential stiffness of an individual contact (to be distunguished from the stiffness of a multicontact interface [22]). The term iωξ acounts for viscous dissipation, where ξ is the drag coefficient. ξ quantifies linear processes in the sense that the stress is proportional to the rate of displacement. No statement is made on the mechanism(s) leading to dissipation. The drag coefficient may be linked to the viscoelastic nature of the materials involved, but also to interfacial processes (as long as these obey linear mechanics). Equation 3 can be inverted as

[2]
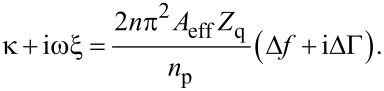


As shown in Equation 2, the complex frequency shift is easily converted to a complex contact stiffness.

Up to now, linear force–displacement relations were assumed. If linearity does not hold, the stress, σ(*t*), is no longer time harmonic. In consequence, there is no complex amplitude, σ_0_, which could be inserted into Equation 3. Importantly, a non-trivial time dependence can be accounted for in an expanded model. As long as stress is periodic with the frequency of the resonator (but of any other shape otherwise), the QCM measures the first Fourier component of σ(*t*). It then follows that [36–37]

[4]
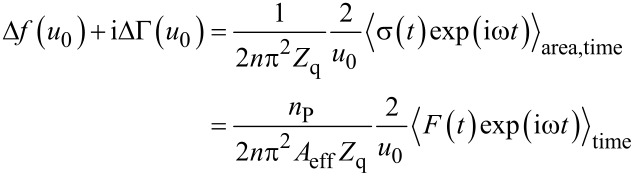


In the 2nd line of Equation 4, stress was replaced by the force at the contacts, *F*(*t*), multiplied by the number density of the contacts, *n*_P_/*A*_eff_. There is a close analogy between Equation 4 and the principle of operation of lock-in amplifiers. Δ*f* and ΔΓ are proportional to the in-phase and the out-of-phase components of the force.

Underlying both Equation 3 and Equation 4 is the small load approximation, which states that the load impedance (often called *Z*_L_, the ratio of σ_0_ and iω*u*_0_) is much smaller than the acoustic shear wave impedance of the crystal, *Z*_q_. The small load approximation holds as long as Δ*f*/*f*_F_ << 1, which is almost always true. If the load is small in this sense, the magnitude of the force is so small that the motion of the resonator surface remains approximately sinusoidal. Put differently, the QCM surface is under displacement control. For that reason, the time average in Equation 4 can be converted to an average over displacement, *u*. Note: In general, the force, *F*, will not only depend on displacement, *u*, but also on the maximum displacement, *u*_0_, and on the frequency, ω. Because the trajectories differ between the two directions of motion, averaging must occur separately for the two directions. The two forces are called *F*_−_(*u*,*u*_0_,ω) and *F*_+_(*u*,*u*_0_,ω), in the following, where the indices “−” and “+” denote movement toward negative and positive *u*. The chain of algebraic conversions must be *F*_±_(*u*,*u*_0_,ω) → *F*(*t*) → {Δ*f*(*u*_0_), ΔΓ(*u*_0_)}. The first entry must be *F*_±_(*u*,*u*_0_,ω), not *F*(*u*). By letting the forces depend on *u*, *u*_0_, and ω, we do not mean to exclude a dependence on velocity. Such a dependence on velocity would implicitly enter *F*_+_(*u*,*u*_0_,ω) and *F*_−_(*u*,*u*_0_,ω) since the velocity itself is a function of *u* and ω, given as iω*u*.

The transformation from *F*_±_(*u*,*u*_0_,ω) to Δ*f*(*u*_0_) and ΔΓ(*u*_0_) take the form [10] of Equation 5 and Equation 6. The frequency shift, Δ*f*, is proportional to a weighted sum of *F*_+_ and *F*_−_. The integrand in Equation 5 is symmetric in *u*/*u*_0_. The integral may therefore be evaluated at positive values of *u*/*u*_0_, only. The term *u*/*u*_0_(1−(*u*/*u*_0_)^2^)^−1/2^ then is positive and takes the role of a statistical weight. Δ*f* is proportional to a weighted average of |*F*_−_ + *F_+_*|, where the weight function has a sharp peak at *u* ≈ *u*_0_. As the relation in the second line of Equation 5 shows, Δ*f* roughly scales as the force at the turning point divided by *u*_0_. The shift in bandwidth is proportional to the difference of *F*_+_ and *F*_−_ (Equation 6). In essence, it is the area under the force–displacement loop and thereforce scales as *u*_0_^2^. The bandwidth is proportional to this area divided by *u*_0_^2^.

[5]
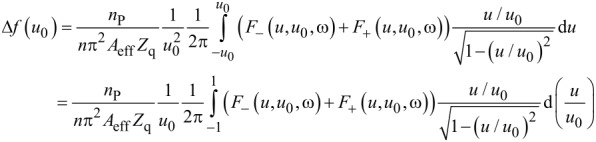


[6]
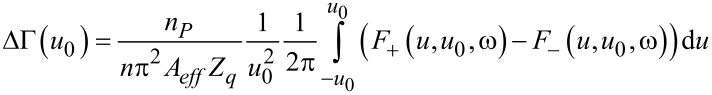


Figure 2C, D illustrates the content of Equation 5 and Equation 6 in graphical form. For viscoelastic contacts, the force–displacement loop is an ellipse. The ratio of force and displacement at the peak (full dots) is independent of amplitude, *u*_0_, and Δ*f* therefore also is independent of *u*_0_. The area inside the friction loop scales as *u*_0_^2^, and ΔΓ therefore also is independent of *u*_0_. This may change, if the force–displacement loop takes some other shape. Figure 2D shows the force–displacement loop according to Cattaneo and Mindlin. For contacts following the CM model, Δ*f* and ΔΓ decrease and increase with amplitude, respectively.

#### Partial slip and its consequences for a QCM experiment: predictions derived from the Cattaneo–Mindlin model and the Savkoor model

In Cattaneo–Mindlin theory, the tangential force, *F_x_*, and the tangential displacement, *u*, are related as [8]

[7]
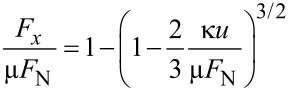


*F*_N_ is the normal force and µ is the friction coefficient in the Coulomb sense. No distinction is made between the static and the dynamic friction coefficient. κ = 2G*a is the contact stiffness in the low-amplitude limit. *a* is the contact radius and G* is an effective modulus. The frequency shift, Δ*f*, is related to the contact stiffness, κ, by Equation 2. G* is the effective modulus, given as

[8]
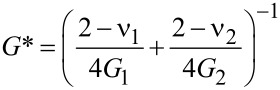


*G* and *v* are the shear modulus and the Poisson ratio, respectively. The indices 1 and 2 label the contacting media. Given that the contact diameter can be estimated to be larger than 1 µm, we ignore the thin films present (SiO_2_, PMMA, gold) and use the same values on both sides.

For the sake of quantitative modeling (see Figure 5 below) we keep the Poisson number fixed at *v*_1_ = *v*_2_ = 0.17 and express the shear modulus as

[9]
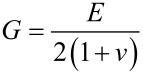


where *E* is the Young’s modulus and *E* is a fit parameter. The contact radius, *a*, is assumed to obey the JKR equation, which is

[10]
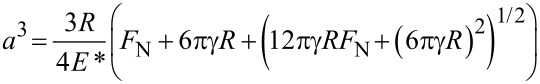


where *R* is the (known) sphere radius, γ is the energy of adhesion and E* is another effective modulus, given as

[11]
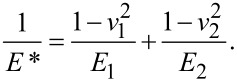


As before, *v*_1_ ≈ *v*_2_ ≈ 0.17 is assumed. Also, *E*_1_ was assumed to be the same as *E*_2_ (*E*_1_ ≈ *E*_2_ = *E*) with *E* a fit parameter. The energy of adhesion, γ, was also a fit parameter. All other parameters were fixed.

Inserting the force–displacement relation from Equation 7 into Equation 5 and Equation 6 and, further, expanding the result to first order in *u*_0_, one finds [10]

[12]
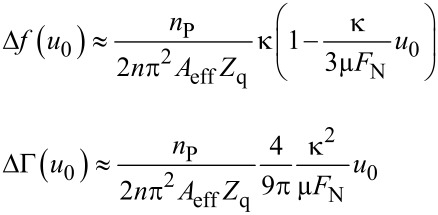


At this point, we slightly extend the CM model by including viscous dissipation. On a heuristic basis, we add a viscous term into ΔΓ which accounts for dissipative processes with a linear dependence on stress:

[1]
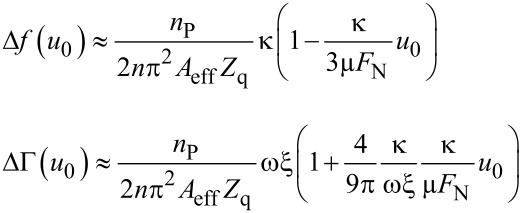


As Equation 1 shows, the Cattaneo–Mindlin theory predicts Δ*f* and ΔΓ to depend linearly on *u*_0_.

Savkoor [14,38] has formulated a modified model of partial slip, which assumes the traction in the sliding zone to be constant with a value of τ_0_, rather than being proportional to the normal stress as in CM theory. The force–displacement relation resulting from the Savkoor model is

[13]
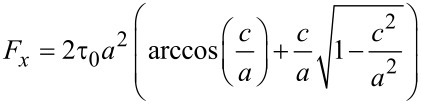


where *a* is the radius of contact and *c* is the radius of the sticking area, given as

[14]
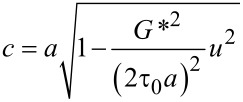


Inserting Equation 13 into Equation 5 and Equation 6 and, further, expanding the result to second order in *u*_0_, one finds [10]:

[15]
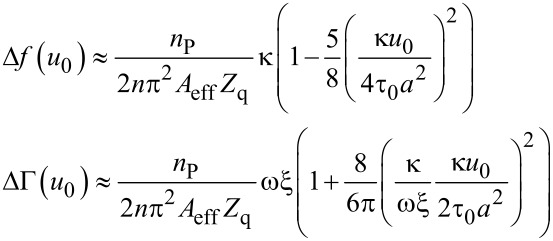


The dependence of Δ*f* and ΔΓ on amplitude is now parabolic, whereas it is linear in the CM model.

#### Experimental details

**Figure 3:**
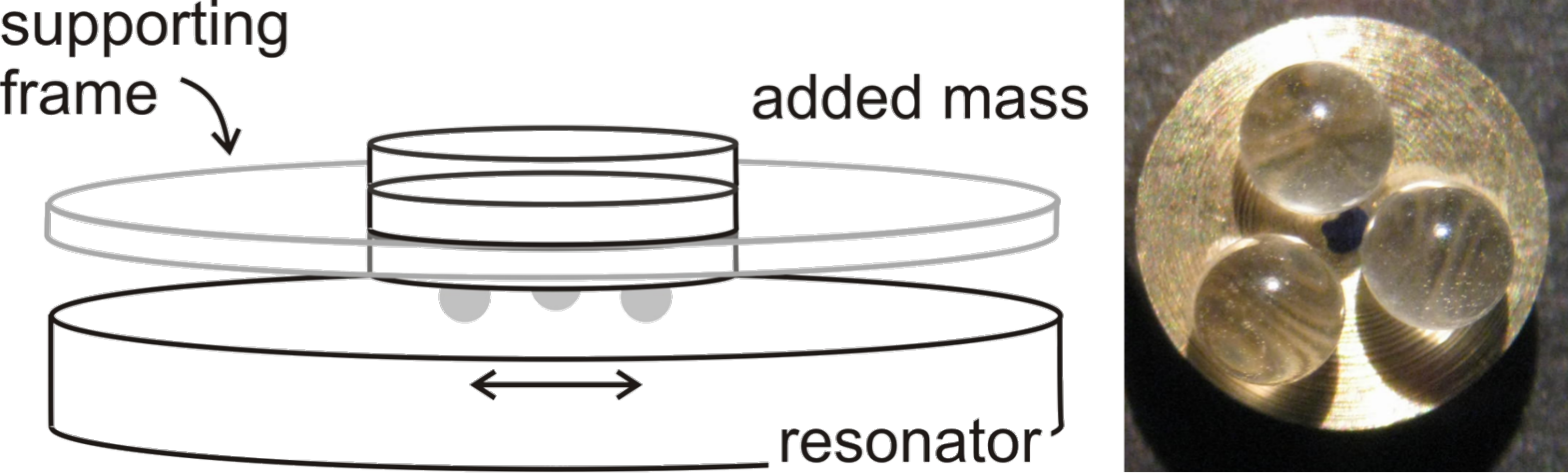
Left: Sketch of the experimental geometry. The contacts are formed between a tripod (center top) and the resonator plate. The three spheres (in grey) carry an equal normal load and experience equal amplitudes of oscillation. The normal force is changed by adding weight on the top. A fixed frame around the tripod ensures that the tripod does not shift when weight is added or removed at the top. Right: Image of a Tripod.

The geometry of the experiment was based on a tripod configuration as shown in Figure 3. Three glass spheres with a diameter of either 2.2 mm or 1.2 mm were glued to a backing plate in the form of an equilateral triangle. The tripod was placed onto the center of the plate, where the distance of the individual contacts to the center was less than 3 mm. The three points of contact experience the same normal force and the same amplitude of motion. The weight of the tripod alone was 0.5 g. Additional weights between 0.5 and 2.5 g were added onto the backing plate, thereby increasing the normal force. There was a frame with a cylindrical hole around the backing plate, which prevented its lateral movement. With this frame in place, the sample did not shift laterally when the weight was added. The frame was essential for obtaining reproducible results.

Shifts of frequency and bandwidth were acquired with impedance analysis. One frequency sweep took about 1 s. Each amplitude ramp consisted of 10–15 steps. All ramps were repeated four times (two increasing and two decreasing ramps). The first ramp often gave results different from the following three ramps. This type of running-in behavior was not further investigated. Most of the time, the data from ramps 2– 4 agreed with each other within the experimental error. In particular, there were no systematic differences between increasing and decreasing ramps. Occasionally, a slow drift was superimposed onto the ramps. Quartz resonators respond to changes in temperature and static stress with slow drifts. Drifts can be reduced by mounting the crystals in the holder one day before the experiment and by controlling temperature, but they cannot be avoided altogether. Experiments were undertaken in ambient air with no additional control of temperature or humidity. For further details on the experiment (on the processing of raw data and on the calculation of the amplitudes, in particular) see [10]. Experiments were carried out with either SiO_2_-coated resonators (purchased from Inficon) or PMMA-coated resonators. The thickness of the spin-cast PMMA layer was 250 nm. Previous experiments did not find evidence of an influence of the thickness of a glassy polymer on the contact stiffness.

All experiments were carried out in both air and water. Deionized water was used throughout, but the water was not degassed. A sample, which had been previously studied in air, was flooded with water. The water level was about 3 mm; however, the exact height was not an important parameter because the QCM only senses the conditions inside the first micron of a liquid sample.

## Results and Discussion

**Figure 4:**
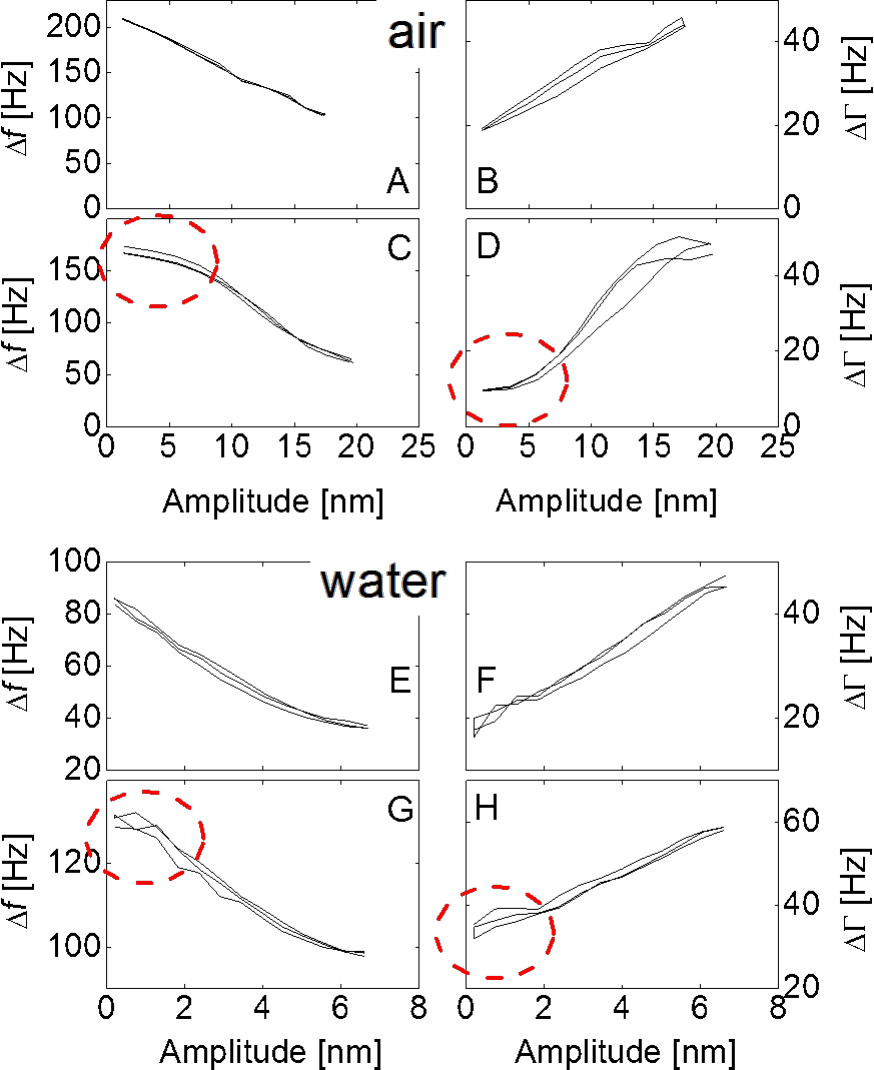
Data traces of frequency shift, Δ*f*, and bandwidth shift, ΔΓ, versus amplitude of oscillation. All data sets contain four amplitude sweeps. Data shown in the four panels at the top and the four panels at the bottom were acquired in air and in water, respectively. In liquids, the maximum achievable amplitude is lower than in air because of damping. Δ*f* and ΔΓ decrease and increase with amplitude, respectively, as is characteristic for partial slip. Panels A, B, E, and F show typical data traces. In these cases, the amplitude dependence is linear. Occasionally, one also finds plateaus at small amplitudes (dashed ellipses in panels C, D, G, and H). In these cases, the edge of the contact sticks at small amplitudes, where the exact conditions, under which such a stick occurs, are unclear. Even in these cases, the frequency–amplitude traces are clearly not parabolic (which should result if the Savkoor model was applicable).

**Figure 5:**
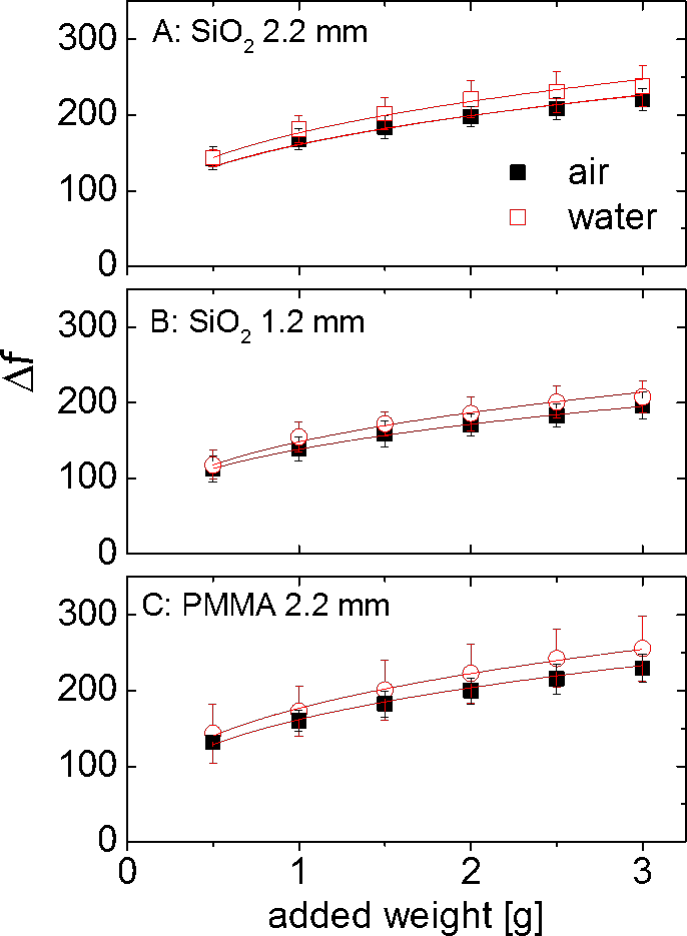
Frequency shifts in the low-amplitude limit obtained on silica surfaces (A and B) and on a PMMA surface (C). All data shown are averages from 9 experiments. Error bars are standard deviations. Dotted lines show JKR fits with parameters as given in Table 1.

Figure 4 shows a number of amplitude sweeps. The four graphs at the top and the four graphs at the bottom display data acquired in air and in water, respectively. Because water damps the crystal’s resonance, the maximum amplitude achieved was 6 nm (compared to an amplitude of ≈20 nm in air). Δ*f* (*u*_0_) is always a decreasing function of amplitude, *u*_0_ (panels on the left-hand side), while ΔΓ increases with *u*_0_ (on the right). Figure 4A,B,E,F displays what was observed most of the time (in >80% of the experiments): Most of the time, Δ*f* and ΔΓ were linear functions of *u*_0_. Occasionally, the data show a plateau at small amplitudes. These plateaus have been discussed in detail in [39]. They can be associated with a critical minimum amplitude for partial slip. A plateau occurred often for the small spheres (diameters <500 µm) examined in [10]. Further discussion is outside the scope of this work. Large spheres were chosen here in order to achieve a linear dependence of Δ*f* and ΔΓ on *u*_0_. If linear behavior is observed, the complex spring constant in the low-amplitude limit is readily extracted from the data by extrapolation (see Figure 5 and Figure 7 below). Likewise, the friction coefficient as derived from the slopes of Δ*f*(*u*_0_) and ΔΓ(*u*_0_) is a robust parameter (see Figure 8 below).

Very rarely, we see an increase of Δ*f* with amplitude (data not shown). This behavior might tentatively be associated with junction growth [12]. Most of the time Δ*f* and ΔΓ decrease and increase with amplitude, characteristic for partial slip.

Figure 5 shows the low-amplitude limits of Δ*f* for the three different configurations studied. Full and open symbols correspond to data taken in air and in water, respectively. The fact that Δ*f*_0_ increases with normal load is easy to understand. With increasing load, the contact radius increases and the contact stiffness increases correspondingly. The dotted lines show an attempt to bring this understanding in line with the known models of contact mechanics. We fitted the data with the JKR model. (The Tabor parameter of the geometry under study is 10, which says that the JKR model should be applied, rather than the DMT model.)

Table 1 shows the derived values of the interfacial energy, γ, and the effective Young’s modulus, *E*. While the values are reasonable, they scatter quite significantly between the different experiments and the different configurations. As far as the interfacial energy, γ, is concerned, part of the problem is that the loads are rather high. A more reliable determination of γ would require more data points close to the point of zero added weight. Clearly, the numbers must be interpreted with some caution. Possible sources of artifacts are roughness, contamination, and of course the idealized assumptions of the model. The high excitation frequency may also play a role. A systematic comparison with the tangential contact stiffness determined at low frequencies would certainly be worthwhile. Unfortunately, such experiments are difficult.

**Table 1:** Fit Parameters for the data in Figure 5.

	air, γ [mN/m]	air, *E* [GPa]	water, γ [mN/m]	water, *E* [GPa]

SiO_2_, 2.2 mm	190 ± 20	100 ± 3	50 ± 40	63 ± 6
SiO_2_, 1.2 mm	30 ± 10	61 ± 2	20 ± 30	47 ± 5
PMMA, 2.2 mm	10 ± 10	70 ± 2	0	45 ± 3

The contact stiffness increased when the sample was immersed in water. Note: The contacts were not broken between the two experiments. Water was admitted to the sample compartment without removing the spheres from the resonator. An increased stiffness in water contradicts intuition insofar, as one would expect the liquid to lower the effective van der Waals attraction. With lowered adhesive forces, the contact area should decrease and the contact stiffness should decrease, in consequence. However, this was not observed. The contact stiffness increased by about 10% in all cases.

**Figure 6:**
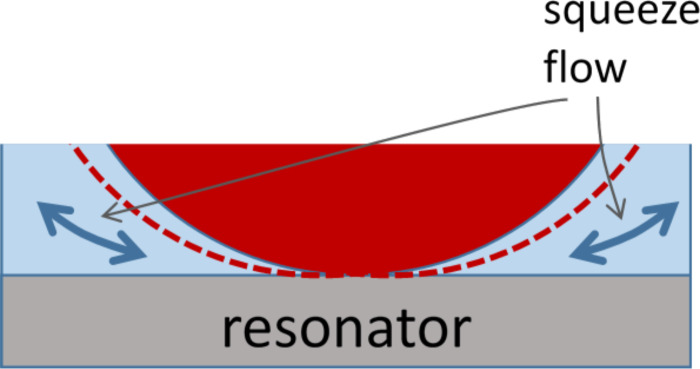
A sketch of an explanation for the increase in MHz contact stiffness when an experiment is undertaken in liquid rather than in air. A rocking motion of the sphere couples to a squeeze flow of liquid in the wedge next to the edge of the contact. Since inertia immobilizes the liquid, there is a significant amount of volume compression. The liquid responds elastically to compression.

At this point, the high frequency of the measurement presumably comes into play in the sense that the small compressibility of the liquid contributes to the contact stiffness. Figure 6 provides a sketch. When the resonator surface oscillates tangentially, the material close to the contact responds with a tangential movement, mostly, but one can also expect a small amount of rotation. The rotational component changes the width of the liquid wedge close to the contact, thereby inducing a squeeze flow of liquid. However, the mass involved in this movement is so large that inertia strongly resists the flow. (The sphere itself is clamped in space for the same reason). Because of inertial clamping, the sphere’s rocking motion compresses the liquid and the liquid responds elastically to compression. The liquid’s high bulk modulus in this way stiffens the contact. Again, this effect is genuinely linked to the experiment occurring at MHz frequency. It will be important when applying this methodology to biomaterials (which are usually studied in the liquid phase). The above interpretation clearly is tentative. Roughness may also play role. When water fills the micro-voids between the two surfaces, this may also increase the elastic stiffness of the contact.

**Figure 7:**
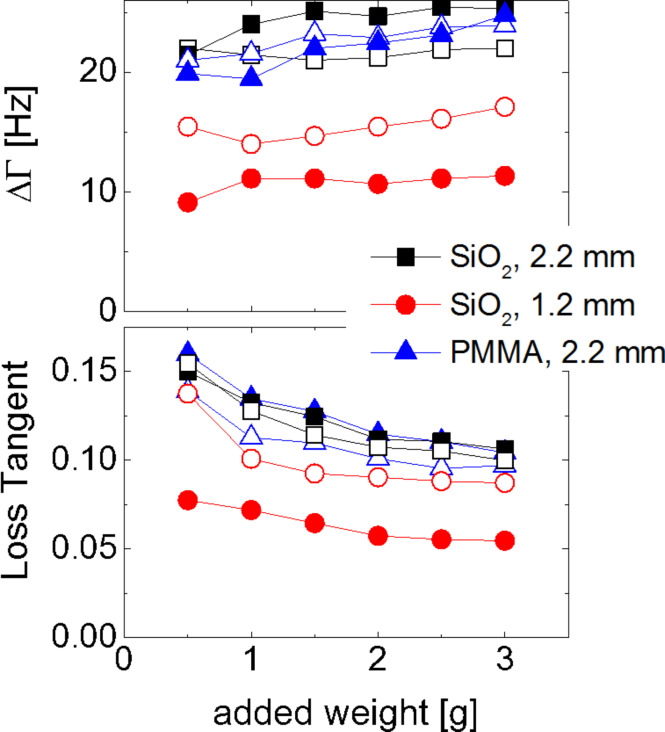
Shift in bandwidth, ΔΓ (top) and loss tangent, ΔΓ/Δ*f*, (bottom) in the low-amplitude limit. Full and open symbols denote measurements in air and water, respectively. The fact that ΔΓ is a constant independent of the normal load, suggests interfacial processes as the source of dissipation. If the dissipation were to occur in the material, one would expect the loss tangent to be constant, rather than ΔΓ itself, because a materials parameter should not depend on the normal load.

Figure 7 addresses the linear components of the dissipative processes, quantified by the low-amplitude limit of ΔΓ, termed ΔΓ_0_. In Figure 7B, Γ_0_ was converted to a loss tangent by taking the ratio of ΔΓ_0_ and Δ*f*_0_. Interestingly, ΔΓ does not increase with normal load in the same way as Δ*f*. It stays approximately constant. For that reason the loss tangent is a decreasing function of normal load. This result implies that the finite values of ΔΓ_0_ should not be viewed as a consequence of viscous dissipation inside the materials involved. If ΔΓ/Δ*f* were a materials parameter, it should not depend on the normal load. Also, a loss tangent of 0.1 would be unreasonably high for fused silica. Rather, these dissipative processes should be attributed to the interface. Linear contributions to the dissipation in contact resonance experiments are well known [8,40]. While the exact nature of these processes would be interesting, the present experiments do not allow for a statement other than that they must be connected to interfacial friction in one way or another.

So far, the discussion has been concerned with linear contact mechanics. The experiment is easy and there are few other techniques that give access to the same data (mostly the AFM and ultrasonic reflectometry). Importantly, the QCM also accesses the (weakly) nonlinear regime and it does so rather easily, as well. As shown in Figure 4, most data sets show a linear dependence of Δ*f* and ΔΓ on *u*_0_. In the following, we use these data to derive the apparent friction coefficient from the slopes, following Equation 1.

**Figure 8:**
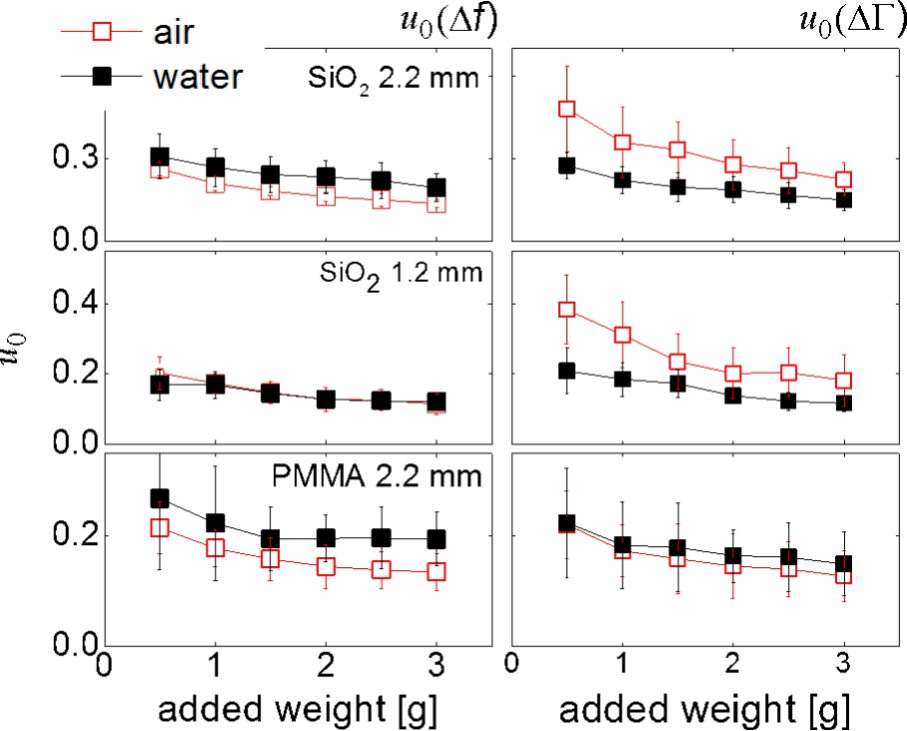
Friction coefficients, µ, obtained by analyzing the slopes in plots of Δ*f* versus *u*_0_ (left) and ΔΓ versus *u*_0_ (right), following Equation 1. The two ways to derive µ should lead to the same values, ideally. Further, µ should be of the order of unity. The decrease of µ with increasing normal force can be explained with an increased contact area and a concomitant increase in the stress concentration at the edge of the contact.

Figure 8 displays these apparent friction coefficients. Firstly, the two ways to derive the friction coefficient (from Δ*f*(*u*_0_) and ΔΓ(*u*_0_)) give reasonable agreement with each other. Secondly, the friction coefficients that result are in the range known from macroscopic mechanics (that is, on the order of unity). Thirdly, and importantly, the friction coefficients all decrease with normal load. The larger the contact area, the more pronounced is the partial slip. This finding is in line with the treatment of the pulling problem by Gao and Yao referred to in the Introduction. Partial slip occurs if the stress singularity at the edge is strong. The peak stress depends on the ratio of the contact radius to the radius of the crack tip and therefore increases as the normal force becomes larger. A different (but related) explanation builds on nanoscale roughness. Nanoroughness rounds off the stress profile at the edge, which avoids the stress singularity similarly to a finite radius of a crack tip. The load dependence of µ points to yet another benefit of “contact splitting” [40–41]. A large number of small contacts will experience less partial slip (less fretting wear) than a small number of correspondingly larger contacts.

A side remark: The agreement between the two friction coefficients (determined from Δ*f*(*u*_0_) and ΔΓ(*u*_0_)) is better in water than in air. We suspect that capillary forces affect ΔΓ(*u*_0_) stronger than Δ*f*(*u*_0_). A more detailed discussion of the matter would require an extension of the Cattaneo–Mindlin model by specific contributions from different forces. Such an extension is outside the scope of this work, but it is possible. It is even worthwhile, if the role contact mechanics in acoustic sensing shall be expanded.

## Conclusion

Using a QCM-based contact resonance method, the stiffness of sphere–plate contacts was studied at MHz frequencies. The linearcontact stiffness increases with normal load. A fit using JKR theory is possible. The fit parameters are in the expected range, but there is a significant amount of scatter between experiments. A quantitative interpretation must be undertaken with some care. The contact stiffness increases in the presence of a liquid. Possibly, this increase is rooted in a squeeze flow close to the edge of the contact. The loss tangent is of the order of 0.1 and decreases with normal force, *F*_N_. The *F*_N_-dependence suggests that the dissipation is connected to interfacial processes. At elevated amplitudes, it was also observed that there is partial slip. The amplitude dependence of frequency and bandwidth can be fitted with the Cattaneo–Mindlin model, which suggests that the frictional forces are proportional to the normal pressure as in macroscopic friction. The friction coefficients were found to be on the order of unity. The friction coefficients as derived from Δ*f*(*u*_0_) and ΔΓ(*u*_0_) agree with each other reasonably well. The agreement is better in water than in air. Finally, the friction coefficients were found to decrease slightly with increasing normal force (that is, with increasing contact area). This can explained by the finite radius of the crack tip at the edge of the contact or by nanoscale roughness. These effects are most pronounced for the smallest contacts. Contact splitting can lower the amount of partial slip and fretting wear.
